# Additive prognostic value of plasma N-terminal pro-brain natriuretic peptide and coronary artery calcification for cardiovascular events and mortality in asymptomatic patients with type 2 diabetes

**DOI:** 10.1186/s12933-015-0225-0

**Published:** 2015-05-21

**Authors:** Bernt Johan von Scholten, Henrik Reinhard, Tine Willum Hansen, Morten Lindhardt, Claus Leth Petersen, Niels Wiinberg, Peter Riis Hansen, Hans-Henrik Parving, Peter Karl Jacobsen, Peter Rossing

**Affiliations:** Steno Diabetes Center, Niels Steensens Vej 1, Gentofte, 2820 Denmark; Center for Functional and Diagnostic Imaging and Research, Hvidovre Hospital, University of Copenhagen, Compenhagen, Denmark; Frederiksberg Hospital, Frederiksberg, Denmark; Gentofte Hospital, Gentofte, Denmark; Rigshospitalet, Copenhagen, Denmark; University of Copenhagen, Copenhagen, Denmark; The Heart Center, Rigshospitalet, University of Copenhagen, Copenhagen, Denmark; Aarhus University, Aarhus, Denmark

## Abstract

**Background:**

In patients with type 2 diabetes, cardiovascular disease (CVD) is the major cause of morbidity and mortality. We evaluated the combination of NT-proBNP and coronary artery calcium score (CAC) for prediction of combined fatal and non-fatal CVD and mortality in patients with type 2 diabetes and microalbuminuria (>30 mg/24-h), but without known coronary artery disease. Moreover, we assessed the predictive value of a predefined categorisation of patients into a high- and low-risk group at baseline.

**Methods:**

Prospective study including 200 patients. All received intensive multifactorial treatment. Patients with baseline NT-proBNP >45.2 ng/L and/or CAC ≥400 were stratified as high-risk patients (n = 133). Occurrence of fatal- and nonfatal CVD (n = 40) and mortality (n = 26), was traced after 6.1 years (median).

**Results:**

High-risk patients had a higher risk of the composite CVD endpoint (adjusted hazard ratio [HR] 10.6 (95 % confidence interval [CI] 2.4-46.3); p = 0.002) and mortality (adjusted HR 5.3 (95 % CI 1.2-24.0); p = 0.032) compared to low-risk patients. In adjusted continuous analysis, both higher NT-proBNP and CAC were strong predictors of the composite CVD endpoint and mortality (p ≤ 0.0001). In fully adjusted models mutually including NT-proBNP and CAC, both risk factors remained associated with risk of CVD and mortality (p ≤ 0.022). There was no interaction between NT-proBNP and CAC for the examined endpoints (p ≥ 0.31).

**Conclusions:**

In patients with type 2 diabetes and microalbuminuria but without known coronary artery disease, NT-proBNP and CAC were strongly associated with fatal and nonfatal CVD, as well as with mortality. Their additive prognostic capability holds promise for identification of patients at high risk.

## Introduction

In patients with type 2 diabetes, cardiovascular disease (CVD) is the major cause of morbidity and mortality. Compared with subjects without diabetes, the risk of cardiovascular complications is two to four times increased, and is even higher in patients with diabetes and established albuminuria [1, 2].

Several risk scores have been developed to estimate the cardiovascular risk in asymptomatic subjects. The Framingham Risk Score is the most commonly applied global risk score [3]. While proven to be useful to identify subjects at risk, these scoring models fail to identify up to 35 % of future CVD. Moreover, as risk scoring programs are not as predictive in diabetic patients compared to the general population better screening tools are needed for the latter large patient population [4]. The UKPDS Risk Engine is a type 2 diabetes specific risk calculator that is probably the most widely used and which estimates absolute risk of coronary heart disease or stroke using traditional risk factors plus diabetes-specific factors including duration of diabetes and HbA_1c_ [5]. Several studies have examined the validity of the UKPDS risk engine with inconsistent results and revised risk equations have been suggested [6, 7].

Brain natriuretic peptide (BNP) and its cleavage product N-terminal (NT)-proBNP are secreted in response to cardiac haemodynamic stress mediated by volume and pressure overload [8]. BNP exhibits biological activity while NT-proBNP has none, and when it comes to analytical methods the in-vitro stability of BNP is assay-dependent, whereas NT-proBNP is very stable at room temperature and measurement of NT-proBNP is therefore most often used in clinical practice [9].

In a type 2 diabetic population followed for 15 years, we previously identified plasma NT-proBNP levels as a powerful predictor of mortality, independent of urinary albumin excretion rate (UAER) and other risk factors [10]. Eighty percent of patients in the upper NT-proBNP tertile (>103 ng/L) died compared to 30 % in the lower tertile (<41 ng/L; p < 0.001). Other studies have also shown a strong association of NT-proBNP with vascular outcomes [11, 12] and mortality [13] in patients with type 2 diabetes.

The coronary artery calcium score (CAC) is a non-invasive screening tool derived from a computed tomography (CT) scanning [14] that reliably identifies patients at high risk of future adverse cardiac events [15]. CAC reflects the global coronary atherosclerotic burden, and the scoring can be quantified by the Agatston score [16]. Diabetic patients with CAC of 0 have the same risk of CVD events as people without diabetes [17], and CAC > 400 has been defined as representative of severe coronary artery disease (CAD) with high risk of anatomic coronary stenosis as determined by coronary angiography (CAG) [18, 19].

To the best of our knowledge, the prognostic value of a combination of NT-proBNP and CAC has never been examined in patients with type 2 diabetes. To address this issue, we evaluate the joint predictive value of NT-proBNP and CAC for combined fatal and non-fatal CVD, and all-cause mortality, respectively, in patients with type 2 diabetes and microalbuminuria, but without known CAD. Moreover, we evaluate the predictive value of our predefined categorisation of patients into a high- and low-risk group at baseline. The pre-specified study hypothesis was that a screening algorithm based on NT-proBNP and CAC was able to identify asymptomatic patients at high risk. Furthermore, analyses of NT-proBNP and CAC as continuous variables were performed to support the results of the categorical analyses.

## Materials and methods

### Participants and study procedure

**Fig. 1:**
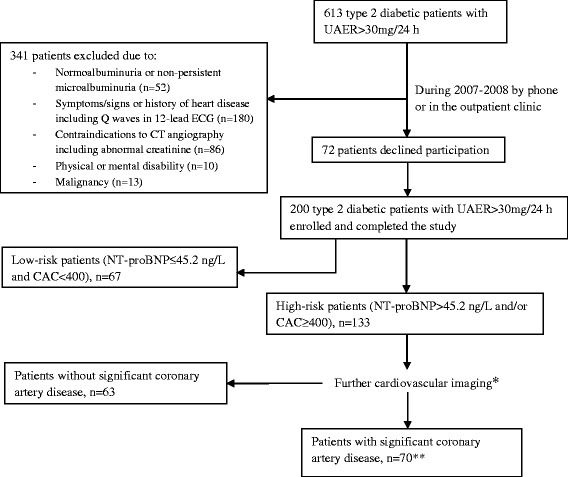
Selection of the study population and algorithm used for risk group assessment with the use of plasma NT-proBNP and coronary artery calcium score (CAC). * (i) patients with P-NT-proBNP >45.2 ng/L underwent myocardial perfusion imaging. Patients with abnormal myocardial perfusion imaging (n = 55) or CAC >100 (n =29) were referred for coronary angiography; (ii) patients with P-NT-proBNP ≤ 45.2 ng/L and CAC 400–1000 underwent CT angiography (n = 20); CT angiography was only used in patients with CAC 400–1000 since severe coronary artery calcifications (CAC > 1000) compromise the validity of CT angiography. Patients with abnormal CT angiography were referred for coronary angiography (n =15) and (iii) patients with P-NT-proBNP ≤ 45.2 ng/L and CAC >1000 underwent myocardial perfusion imaging (n =9). Patients with abnormal myocardial perfusion imaging (n = 6) were referred for coronary angiography.**Significant coronary artery disease was defined as the presence of one or more significant myocardial perfusion defects on myocardial perfusion imaging, and/or one or more significant major epicardial coronary artery stenosis at coronary angiography

At Steno Diabetes Center, we identified from January 2007 to February 2008 a cohort of 200 outpatients with type 2 diabetes treated in a secondary care setting. All patients received treatment with intensive multifactorial intervention constituting of strict glycaemic, lipid and blood pressure control, as well as antithrombotic therapy and lifestyle modification according to the Steno-2 study [20]. Patients were aged between 20 and 70 years, with the ability to understand and give informed consent. Patients were included if they met the following inclusion criteria’s: 1) outpatients with type 2 diabetes defined according to WHO criteria’s; 2) no history of CAD or other cardiac diseases and without any symptoms from the heart, assessed from patient files and thorough interviews and questionnaires; and 3) persistent (two out of three consecutive measurements) UAER > 30 mg/24 h. Written invitation was sent to 613 consecutive patients (69 % males and a mean (standard deviation [SD]) age of 47 (8) years). A total of 72 patients refused to participate. Furthermore, patients (n = 341) were excluded (either by phone interview or after examination in the outpatient clinic) if one or more of the following characteristics were present: 1) normal UAER or non-persistent elevated UAER (n = 52); 2) symptoms/signs or history of heart disease including Q waves in 12-lead ECG (n = 180); 3) relative contraindications to CT angiography (CTA) or CAG, including abnormal plasma creatinine levels (n = 86); 4) physical or mental disability (n = 10); or 5) malignancy (n = 13). Thus, the final study population included 200 patients. A detailed flow chart of the selection of the study population is shown in Fig. 1. The baseline data has previously been presented [8].

The a priori sample size calculation was based on the assumption that 20-30 % of the patients would experience a cardiovascular event during 5 years of follow-up. The treating physicians were blinded to the predictor variables NT-proBNP and CAC during the follow-up period. This study complies with the Declaration of Helsinki, the research protocol was approved by the local ethics committee and all patients gave written informed consent.

### Biochemical analyses and other

NT-proBNP was measured in all patients and analysed by an immunoassay as previously described [10]. UAER was measured in 24 h urine collections by an enzyme immunoassay [21]. Current smoking was defined as one or more cigarettes/cigars/pipes a day. The estimated Glomerular Filtration Rate (eGFR) was calculated using the Chronic Kidney Disease Epidemiology Collaboration (CKD-EPI) equation [22]. Transthoracic echocardiography was performed using a Philips IE 33 machine (Phillips Medical Systems, Best, The Netherlands). Simpson’s apical biplane method was used to evaluate left ventricle ejection fraction (LVEF), as previously described [23].

### Coronary artery calcium score

Determination of CAC was performed during a single breath hold using a 16 multidetector-row CT scanner with 3-mm-slice thickness (Philips Precedence MX 8000 IDT 16 slice; Philips Medical Systems, Best, The Netherlands). Quantification of Agatston CAC [16], including intimal and medial calcification in the left main, left anterior descending artery, circumflex artery and right coronary artery, was performed on a separate workstation with dedicated software (Heartbeat-CS, EBW; Philips Medical Systems) and summed to provide a total CAC for each participant.

### Stratification into risk groups

Patients were stratified into high-risk and low-risk groups according to CAC and the median NT-proBNP of the first 50 patients examined in the study [8]: (i) NT-proBNP >45.2 ng/L = high-risk patients (*n* = 104); (ii) NT-proBNP ≤45.2 ng/L and CAC ≥ 400 = high-risk patients (*n* = 29) and (iii) NT-proBNP ≤45.2 ng/L and CAC < 400 = low-risk patients (*n* = 67). High-risk patients (n = 133) were further examined by myocardial perfusion scintigraphy imaging (MPI), CTA or CAG according to the algorithm shown in Fig. 1, which has previously been described in details [8]. Based on these additional examinations, 70 high-risk patients without any cardiovascular symptoms was exposed to have significant CAD, defined as the presence of one or more significant myocardial perfusion defects on MPI, and/or one or more significant major epicardial coronary artery stenosis at CAG, and 63 of the high-risk patients had no significant CAD.

### Follow-up

At 1st of January 2014, we traced all patients through the Danish National Death Register and the Danish National Health Register. For deceased patients, we obtained information on the date and cause of death. All deaths were classified as CVD unless an unequivocal non-CVD cause was established. Information about hospital admission including non-fatal CVD was obtained from the Danish National Health Register.

The predefined primary endpoint was the combination of cardiovascular mortality, non-fatal myocardial infarction (ICD-10 codes I20 to I25), stroke (ICD-10 codes I61or I63), ischaemic cardiovascular disease (ICD-10 codes I70), and heart failure (ICD-10 code I50). For participants who experienced multiple endpoints, the analysis included only the first event.

The secondary endpoint was all-cause mortality. No participants were lost to follow-up.

### Statistical analyses

Logarithmic transformation was performed to achieve normal distribution for NT-proBNP, CAC and UAER. All continuous variables are given as medians with interquartile range (IQR) and the categorical variables are reported as total numbers with corresponding percentages. Differences between groups were calculated using Mann–Whitney *U* Test for continuous and *χ*2 for categorical variables.

First, we used Kaplan-Meier survival function estimates and the log-rank test to estimate and compare incidence rates by risk group. Next, we used Cox proportional hazards analyses to compute hazard ratios (HRs) and 95 % confidence intervals (CIs) for 1 SD increase of the log transformed values. We adjusted for sex, age, LDL and HDL cholesterol, smoking, HbA1c, eGFR, systolic blood pressure and UAER. In fully adjusted models with known risk factors for CVD, we additionally included NT-proBNP and/or CAC. We evaluated the additive vs. synergistic effects of NT-proBNP and CAC on the endpoints by using appropriate interaction terms and likelihood-ratio tests. The additive predictive value of both NT-proBNP and CAC were evaluated with the use of relative integrated discrimination improvement (rIDI) based on the Cox models. In addition, the continuous net reclassification index (cNRI), with 5 % risk reclassification as cut-point between models, was calculated based on logistic models. To facilitate interpretation of the results, we also computed 5-year absolute risk of the composite CVD endpoint and all-cause mortality associated with CAC at different levels of NT-proBNP from the adjusted Cox models.

A two-tailed p-value < 0.05 was considered significant. Statistical analyses were performed using SPSS for Windows, version 20.0 (SPSS, Chicago, IL) and SAS software (version 9.3 and Enterprise Guide version 5.1; SAS Institute, Cary, NC).

## Results

### Patient characteristics and risk groups

The study population (n = 200) included 76 % men, median (IQR) age was 60 (54–65) years, and median (IQR) of NT-proBNP and CAC was 48.7 (18.6–95.0) ng/L and 183 (6–604), respectively. Table 1 lists clinical characteristics of all patients and the comparisons between the high-risk and low-risk group. High-risk patients were older, had longer known diabetes duration, lower eGFR and total cholesterol levels, and were more frequently on beta-blocker treatment than patients classified as low-risk (p ≤ 0.048).

**Table 1:** Baseline clinical characteristics of all patients, and low- versus high-risk patients

	All patients	Low-risk patients	High-risk patients	p-values
	(*n* = 200)	(*n* = 67)	(*n* = 133)	
Male, *n* (%)	152 (76)	50 (75)	102 (77)	0.75
Age (years)	60 (54–65)	55 (47–61)	62 (59–67)	<0.0001
Duration of diabetes (years)	12 (7–18)	8 (4–14)	14 (9–19)	<0.0001
Body mass index (kg/m^2^)	31.4 (28.5-35.6)	31.7 (28.7-35.9)	31 (28–36)	0.66
HbA_1c_ (%)	7.6 (6.9-8.8)	7.9 (7.0-9.0)	7.5 (6.8-8.8)	0.09
HbA_1c_ (mmol/mol)	60 (52–73)	63 (53–75)	69 (51–73)	0.09
UAER (mg/24 h)	103 (39–230)	105 (44–194)	97 (38–97)	0.81
eGFR (CKD-EPI)	91.0 (76.0-102.0)	100.0 (86.0-107.0)	86.0 (74.0-97.5)	<0.0001
Systolic blood pressure (mmHg)	129 (118–142)	130 (116–140)	129 (119–142)	0.90
Total cholesterol (mmol/L)	3.8 (3.2-4.5)	4.0 (3.4-4.8)	3.8 (3.1-4.4)	0.048
LDL cholesterol (mmol/L)	1.7 (1.3-2.2)	1.8 (1.2-2.4)	1.7 (1.3-2.1)	0.33
HDL cholesterol (mmol/L)	1.1 (0.9-1.4)	1.1 (1.0-1.3)	1.1 (0.9-1.4)	0.35
Current smoker, *n* (%)	59 (30)	18 (27)	41 (31)	0.56
Left ventricle ejection fraction (%)	60 (57–63)	60 (57–62)	60 (57–63)	0.33
History of stroke, *n* (%)	19 (10)	4 (6)	15 (11)	0.19
NT-proBNP (ng/L)	48.7 (18.6–95.0)	15.3 (9.3–26.3)	77.1 (48.7–141.7)	-
Coronary artery calcium score	183 (6–604)	7 (0–104)	417 (80–963)	-
Treatment with:				
Oral antidiabetic, *n* (%)	170 (85)	57 (85)	113 (85)	0.98
Insulin, *n* (%)	124 (62)	38 (57)	86 (65)	0.28
RAAS blockade, *n* (%)	196 (98)	65 (97)	131 (98)	0.48
Statin, *n* (%)	189 (95)	62 (93)	127 (95)	0.39
Aspirin, *n* (%)	183 (92)	58 (87)	125 (94)	0.08
Beta-blocker, *n* (%)	27 (14)	2 (3)	25 (19)	0.002
Calcium channel blockers, *n* (%)	80 (40)	21 (31)	59 (44)	0.08
Diuretics, *n* (%)	128 (64)	35 (52)	93 (70)	0.014

High-risk patients = patients with plasma NT-proBNP levels >45.2 ng/L or plasma NT-proBNP levels ≤45.2 ng/L and coronary artery calcium score ≥400, all other low-risk patients

P-values reflect comparison between high- and low-risk patients

Data are expressed as median (interquartile range) or number of patients (%)

UAER: urinary albumin excretion rate; RAAS: renin-angiotensin-aldosterone system

In addition to oral antidiabetic medications and insulin most patients were treated with cardiovascular medications: statins (95 %), aspirin (90 %), renin-angiotensin-aldosterone system blockade (98 %), diuretics (64 %), calcium channel blockers (40 %) and beta-blockers (14 %).

### Incidence of endpoints

Median follow-up was 6.4 (range: 5.8-7.2) years for the survivors, 6.2 (0.8-7.2) years for the patients reaching the CVD endpoint, and 3.8 (0.3-6.8) years for the non-survivors. During this period, 40 patients experienced at least one CVD endpoint and 26 patients died.

Eleven CVD events were fatal and 29 were non-fatal events leading to hospital admission, including 2 fatal and 3 non-fatal cases of acute myocardial infarction, 3 non-fatal strokes, 1 fatal and 19 non-fatal cases of ischaemic cardiovascular disease, 6 sudden and otherwise unexplained deaths, and 2 fatal and 4 non-fatal cases of heart failure. Of the 26 deaths, 10 were CVD-related, 9 were cancer-related and 7 were due to other causes.

### Risk prediction in high-risk versus low-risk group

Of the 40 patients with a CVD endpoint, 38 were in the high-risk and two in the low-risk group. The 26 deceased patients comprised 24 in the high-risk and two in the low-risk group.

**Fig. 2:**
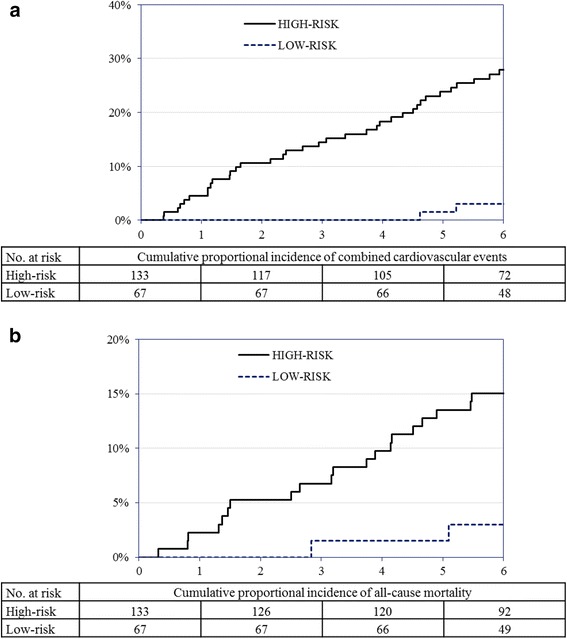
**a** Kaplan-Meier survival function estimates for risk of combined cardiovascular events by categorisation into low- and high-risk at baseline. Hazard ratio 11.4 (95 % confidence interval 2.7-47.3); p < 0.0001. **b** Kaplan-Meier survival function estimates for risk of all-cause mortality by categorisation into low- and high-risk at baseline. Hazard ratio 6.4 (95 % confidence interval 1.5-27.1); p = 0.004

In a Cox regression analysis, patients classified at high-risk at baseline had a significantly higher risk of the composite CVD endpoint compared to low-risk patients (unadjusted HR 11.4 (95 % CI 2.7-47.3); p = 0.001, Fig. 2a; adjusted HR 11.8 (95 % CI 2.7-52.8); p = 0.002). Similarly, high-risk patients had a significantly higher risk of all-cause mortality in both unadjusted (HR 6.4 (95 % CI 1.5-27.1); p = 0.012, Fig. 2b) and adjusted (HR 5.9 (95 % CI 1.3-27.0); p = 0.022) models.

### Risk associated with NT-proBNP and CAC in continuous analyses

In adjusted continuous analysis, higher NT-proBNP was a strong predictor of the composite CVD endpoint and all-cause mortality (p ≤ 0.002; Table 2). In the adjusted model including NT-proBNP, higher age and LDL were also predictors of the composite CVD endpoint (p ≤ 0.047), while only smoking was a predictor of all-cause mortality (p = 0.021).

**Table 2:** Hazard ratios for a 1 SD increase of the log transformed values of NT-proBNP and coronary artery calcium score for fatal and nonfatal cardiovascular events and all-cause mortality

Label		Cardiovascular events	All-cause mortality
Number of events (%)		40 (20)	26 (13)
NT-proBNP	Unadjusted	1.9 (1.4–2.7)^c^	2.2 (1.4–3.4)^c^
	Adjusted	1.9 (1.3–2.7)^b^	2.2 (1.4–3.6)^b^
	Fully adjusted	1.7 (1.1–2.5)^a^	1.9 (1.2-3.2)^a^
Coronary artery calcium score	Unadjusted	3.6 (2.0–6.4)^c^	3.4 (1.7–6.8)^c^
	Adjusted	3.7 (1.9–7.4)^c^	2.9 (1.4–6.3)^b^
	Fully adjusted	3.4 (1.7–6.7)^c^	2.6 (1.2–5.6)^b^

Values are hazard ratios (95 % confidence intervals) and represent a 1 SD increase of the log transformed values of NT-proBNP and coronary artery calcium score

Adjusted models include sex, age, LDL and HDL cholesterol, smoking, HbA1c, eGFR, systolic blood pressure and urinary albumin excretion rate. Fully adjusted models additionally include coronary artery calcium score and NT-proBNP mutually

Significance of the hazard ratios: ^a^P < 0.05, ^b^P <0.01, ^c^P <0.0001

Also, adjusted continuous analysis of CAC revealed a strong independent prediction for risk of both the composite CVD endpoint and all-cause mortality (p ≤ 0.006; Table 2). In the adjusted model including CAC, no other variables were predictive of the composite CVD endpoint or all-cause mortality (p ≥ 0.12).

Both NT-proBNP and CAC remained significantly associated with risk of CVD and all-cause mortality in fully adjusted models mutually including both risk factors (p ≤ 0.022; Table 2). Addition of the interaction term between NT-proBNP and CAC was non-significant in relation to both CVD events (p = 0.90) and all-cause mortality (p = 0.31).

In adjusted analysis the rIDI for NT-proBNP in relation to the CVD endpoints was 28.7 % (p = 0.005), and 62.1 % (p = 0.01) for all-cause mortality. In similar analyses for CAC, the rIDI was 94.4 % (p < 0.0001) for the CVD endpoints, and 142.5 % (p < 0.0001) for all-cause mortality. Addition of NT-proBNP to the adjusted model had significant impact on cNRI for both CVD endpoints and all-cause mortality (0.36, p = 0.008 and 0.37, p = 0.02; respectively). Addition of CAC to the adjusted model had significant impact on CVD endpoints (0.57, p < 0.0001) and borderline impact on all-cause mortality (0.29, p = 0.055).

**Fig. 3:**
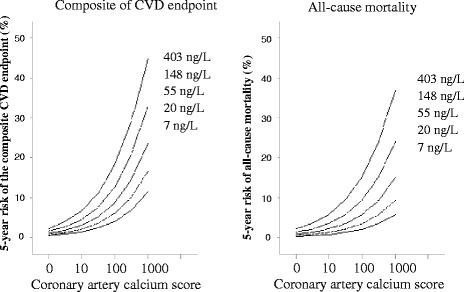
The continuous risk functions cover the 5^th^ to 95^th^ percentile interval of the coronary artery calcium score and correspond to levels of NT-proBNP at 7, 20, 55, 148, 403 ng/L (approximate the 5^th^, 25^th^, 50^th^, 75^th^ and 95^th^ percentiles of the distribution). Risk functions were fitted by Cox regression with adjustment for sex, age, smoking, LDL and HDL cholesterol, HbA1c, eGFR, systolic blood pressure and urinary albumin excretion rate at baseline

Figure 3 shows the fully adjusted 5-year absolute risk for CVD endpoints and all-cause mortality according to NT-proBNP and CAC.

### Additional analyses

At baseline, high-risk patients were subdivided into two groups based on further examinations (Fig. 1): High-risk patients with significant CAD (n = 70) and high-risk patients without CAD (n = 63). In additional analyses comparing each of these two high-risk groups to the low-risk group, both high-risk groups had an elevated risk of the composite CVD endpoint (adjusted HR ≥4.0; p ≤ 0.025), while risk of all-cause mortality was only elevated for the high-risk patients without CAD compared to low-risk patients (adjusted HR 7.7; p = 0.022).

When comparing the two high-risk groups, the high-risk patients with CAD at baseline had an elevated risk of the composite CVD endpoint compared with the high-risk patients without CAD (adjusted HR 2.8; p = 0.012). However, the risk of all-cause mortality was comparable between the two high-risk groups (p = 0.55).

In analyses including all 200 patients, additionally inclusion of LVEF in the adjusted models revealed that both NT-proBNP (adjusted HR 1.6; p = 0.027) and CAC (adjusted HR 3.3; p = 0.001) remained predictive of CVD endpoints. Similarly, both NT-proBNP (adjusted HR 1.8; p = 0.026) and CAC remained predictors of all-cause mortality (adjusted HR 2.9; p = 0.013). In neither of these models, LVEF was a significant predictor (p ≥ 0.08).

Similar models including history of stroke (n = 19) in the multivariate adjustment, revealed that NT-proBNP (adjusted HR 1.7; p = 0.017) and CAC (adjusted HR 2.9; p = 0.02) remained predictive of CVD endpoints. Also for all-cause mortality, both NT-proBNP (adjusted HR 2.2; p = 0.001) and CAC (adjusted HR 3.0; p = 0.009) remained predictive. History of stroke was a predictor of CVD endpoints (HR 3.3; p = 0.017), but not of all-cause mortality (p > 0.27).

## Discussion

In this prospective study of asymptomatic patients with type 2 diabetes and microalbuminuria, without known CAD, risk stratification with NT-proBNP and CAC, alone and in combination, predicted CVD and all-cause mortality on top of established risk factors. According to our previously defined risk criteria based on NT-proBNP and CAC, 24 of 26 deaths occurred in the high risk group during 6.1 years of follow-up and in these patients the adjusted HR for fatal or nonfatal CVD was 10.6 (95 % CI 2.4-46.3). Both risk markers added independent predictive value in the assessment of risk for future events. Identifying diabetic subjects at risk of CVD and death at an early stage and initiating more aggressive therapy in these individuals could improve outcome.

We demonstrated that patients stratified into a high-risk group (NT-proBNP ≥45.2 ng/L and/or CAC ≥ 400) had a significantly higher risk of CVD events and mortality compared to low-risk patients. Findings were similar when comparing each of the high-risk groups with the low-risk group. When comparing the high-risk patients with significant CAD with the high-risk group without CAD, the former had a higher risk of CVD endpoints, while there was no difference in all-cause mortality between these groups (presumably explained by the high fraction of cancer-related deaths). Patients stratified as low-risk had a strikingly low rate of CVD and fatalities.

In patients with type 2 diabetes, elevated UAER is considered to be the most consistent independent predictor of adverse outcomes [24]. Indeed, in more recent prospective studies in patients with type 2 diabetes, NT-proBNP has been reported to be a strong and independent predictor of mortality [13] and cardiac events [11, 25]. In addition, NT-proBNP has been shown to be a strong risk predictor of CVD events in patients with stable CVD [26]. In agreement with this contention, our study showed that higher NT-proBNP was predictive of CVD and mortality even after adjustment for CAC and traditional CVD risk factors.

With regard to CAC, several studies in patients with type 2 diabetes have demonstrated CAC to be a useful risk marker for CVD events and mortality [15, 27–31]. An extensive high CAC (>600) has been shown to be related to high risk of developing chronic CAD-related events; whereas acute CAD-related events mainly occurred in subjects with mild and moderate CAC score (1–600) [32]. These findings were confirmed in our study in patients with albuminuria, where a higher CAC was associated with increased risk of CVD events and mortality even in fully adjusted models.

While CAC is strongly correlated with a patient’s underlying coronary atherosclerotic plaque burden, high levels of NT-proBNP are associated with depressed systolic function and diastolic dysfunction. Thus, NT-proBNP may provide unique risk information beyond the extent of coronary atherosclerosis defined by CAC, which indeed was the rationale of our risk stratification model performed at baseline. After 6 years of follow-up, we found this model to be very potent for prediction of CVD and mortality. The combination of the two measures has previously been investigated in two large cohorts of non-diabetic subjects and here demonstrated improved risk prediction for CVD and mortality in comparison to traditional cardiovascular risk factors [33, 34]. However, to our knowledge, we are the first to investigate the combined prognostic effect of NT-proBNP and CAC in patients with type 2 diabetes and we demonstrated an additive effect of these two risk factors. Therefore, we consider the combination of NT-proBNP and CAC to provide unique risk information in asymptomatic subjects with type 2 diabetes. The usual risk stratification measures in symptomatic patients consists of a combination of echocardiography and CAG, while our stratification model provided prognostic information for asymptomatic patients, but non-invasively and at a much lower cost. Although the identification of asymptomatic patients with type 2 diabetes at high risk of adverse outcomes may be accomplished by our simple screening algorithm, a major concern is how to eliminate or at least minimize the high risk burden. Accordingly, it remains to be proven that implementation of more aggressive medical management in these patients with elevated NT-proBNP and/or CAC improves clinical outcome. However, a randomized controlled study in 300 patients with type 2 diabetes and elevated NT-proBNP (>125 pg/ml) demonstrated that accelerated up-titration of renin-angiotensin-aldosterone system- and beta-blockers to maximum tolerated dosages was an effective and safe intervention for the primary prevention of CVD events. The study did not document a decrease in NT-proBNP concentrations in the treatment group [35]. Treatment of patients with increased CAC scores with lipid-lowering drugs has also been shown to reduce subsequent CVD [36], while another study did not find intensive lipid-lowering treatment to prevent progression of CAC [37]. Thus, more intervention studies are needed in order to clarify therapies with best impact to decrease/prevent CVD. While the optimal medical strategy is not yet fully elucidated in these high-risk patients, it is tempting to speculate that early invasive therapy with CAG and coronary revascularization would be useful. However, the effect of coronary revascularization in asymptomatic patients is controversial and such procedures are currently not recommended [38].

It should be acknowledged that assessment of NT-proBNP is feasible using a simple blood test, while quantification of CAC requires CT imaging, which includes radiation exposure to the patient and is of much higher cost. Therefore, selection of subjects for CT imaging should be performed carefully, albeit that this examination is rapid (<5 min) and the radiation dose is low (1.5 mSv) compared to, e.g. CTA (12 mSv). These cost and safety issues underscore the need for further investigations to maximize potential benefits of screening algorithms and aggressive therapy in high-risk patients. Given that multifactorial treatment is already applied, this algorithm provides prognostic information, but cannot guide additional treatment decisions. For a patient where multifactorial intervention is not initiated the algorithm would strengthen the recommendation for such intervention. New intervention studies are needed to clarify additional treatment options to mitigate the increased risk of CVD and mortality in type 2 diabetes. Considerations might include new therapeutic options, lower targets or increased doses of already applied treatments.

### Strengths and limitations

Even though a limitation of our study is the relatively low number of patients and consequent limited amount of study endpoints making the conclusions not very robust, we found our risk stratification model to be very efficient for identification of subjects at risk of CVD. Out of 40 fatal and nonfatal CVD endpoints, only two low-risk patients experienced an event, which was nonfatal in both cases, and only two low-risk patients died during six years of follow-up, both from non-cardiovascular causes. Our initial division of the study cohort into high- or low risk patients might have provided changes in treatment and intervention, and therefore the estimate of risk in the high-risk group is likely to be conservative. However, despite this, risk was still elevated in the high-risk group. The strength of the study is the prospective design and inclusion of asymptomatic patients with type 2 diabetes and microalbuminuria, which is an established predictor of adverse outcome.

## Conclusion

In asymptomatic patients with type 2 diabetes and microalbuminuria without known CAD, risk stratification with NT-proBNP and CAC was strongly associated with fatal and nonfatal CVD, and all-cause mortality, respectively. While both NT-proBNP and CAC were strong risk factors, their additive prognostic effect holds promise for identification of patients at high-risk.
